# Endovascular intervention in the maintenance and rescue of paediatric arteriovenous fistulae for hemodialysis

**DOI:** 10.1007/s00467-018-4143-8

**Published:** 2018-11-27

**Authors:** Petrut Gogalniceanu, Sam Stuart, Narayan Karunanithy, Nicos Kessaris, Derek Roebuck, Francis Calder

**Affiliations:** 1 Guy’s and St. Thomas’ NHS Foundation Trust, London, UK; 2 Great Ormond Street Hospital for Children NHS Foundation Trust, London, UK; 3 King’s College London, London, UK

**Keywords:** Haemodialysis, Arteriovenous fistulas, Vascular access, Angioplasty, Endovascular

## Abstract

**Background:**

Arteriovenous fistulae (AVF) provide superior primary vascular access for children on chronic dialysis compared to central venous catheters (CVC). However, AVFs inevitably develop complications and will require some intervention to maintain long-term functional patency.

**Methods:**

We report an ‘endovascular-first’ approach to the maintenance and rescue of paediatric AVFs. Thirty interventions targeting 46 lesions in 18 children (median age 11 years [range 5–17]) were performed. Sixty-eight percent of the AVFs were brachio-cephalic fistulae, 26% brachio-basilic fistulae and 5% radio-cephalic fistulae. Immediate functional success was 86% with good dialysis adequacy (mean urea reduction ratio > 70%) at 3 months post procedure.

**Results:**

There was one significant complication, consisting of an AVF rupture which was managed with a covered stent.

**Conclusions:**

Repeated interventions may be necessary to maintain AVF patency and avoid central venous catheters. This is the largest series reported to date.

## Introduction

Arteriovenous fistulae (AVF) are recognised as the gold standard type of vascular access for hemodialysis (HD) in adults [1, 2]. A fistula-first policy for paediatric hemodialysis has been advocated since 2005 [3], yet international registries show that the majority of children are maintained on central venous catheters (CVC) [4, 5]. Hemodialysis via a functional AVF provides superior clinical outcomes compared to hemodialysis using a CVC, including quality of dialysis, lower infection rates and reduced hospitalisation [6]. However, AVFs also develop complications such as stenosis and thrombosis, requiring intervention to extend their functional patency. Fistula surveillance programmes, as recommended by the Kidney Disease Outcomes Quality Initative (KDOQI) guidelines [7], frequently identify AVF-related complications which can be managed by endovascular and open surgical methods. Perhaps due to the low volume of paediatric AVFs in use [8, 9], little has been written regarding the efficacy of endovascular intervention in children with AVF complications.

## Aim

This case series aims to determine the outcome of an ‘endovascular-first’ approach to managing malfunction in paediatric AVFs for haemodialysis. It is the largest series to date.

## Method

A retrospective review of endovascular intervention for paediatric AVFs from 2005 to 2016 years was undertaken. Data were collected from two paediatric tertiary centres (Great Ormond Street Hospital and Evelina Children’s Hospital) London, UK. Medical and radiological case records were retrieved from the dialysis units’ computer databases and radiology archives. Participants were included if they underwent endovascular intervention for acute AVF thrombosis or clinically significant consequences of AVF stenoses affecting the quality of haemodialysis.

The primary end-point was immediate success of the endovascular procedure, defined as the ability to undergo effective hemodialysis through a patent AVF in the first 12 h following intervention. The secondary end-point was quality of dialysis at 3 months following endovascular intervention. Complications of angioplasty were also reported.

The angiographic images (‘fistulograms’) of the AVFs were independently reviewed by two consultant interventional radiologists, in order to determine lesion location and morphology. The severity of stenoses was calculated as a ratio of the maximum internal stenotic luminal diameter compared to the maximum internal luminal diameter in a normal segment of adjacent upstream vessel. The lesions’ anatomical location was described using the classification system outlined in Table 1.

**Table 1:** Anatomical classification of AVF stenotic lesions

Class	Anatomy	Definition
I	Anastomotic	At the arteriovenous anastomosis
II	Post-anastomotic	Within a vascular segment 3 cm downstream from the anastomosis
III	Mid-vessel	Functional needling segment of the AVF, that is 3 cm downstream to the AV anastomosis and proximal to the ‘swing point’; these include the mid-forearm, mid-humeral or upper humeral areas.
IV	Swing point	Vascular segment crossing from a superficial to a deep venous system, such as the cephalic vein in the delto-pectoral groove or basilica vein confluence with brachial veins
V	Central	Intra-thoracic vascular segment, which includes the SVC, IVC, brachiocephalic and subclavian veins

*AVF* arteriovenous fistulae, *SVC* superior vena cava, *IVC* inferior vena cava

## Results

The study reviewed all endovascular procedures undertaken between 2005 and 2016. Eighteen children were included in the study. All the interventions were performed as the first treatment modality for the AVF dysfunction.

### Demographics

At the time of intervention, the median age was 11 years (*n* = 30; age range 5–17) with a median weight of 27 kg (range 20–66). Sixty-eight percent of the AVFs were brachio-cephalic fistulae, 26% brachio-basilic fistulae and 5% radio-cephalic fistulae. Predominantly, these were positioned in the non-dominant, left limb (74%). All fistulae were formed for hemodialysis access for chronic kidney disease only. The cause of chronic kidney disease (CKD) stage V was known in 78% of patients (*n* = 14). A range of aetiologies were identified, with the majority being attributed to focal segmental glomerulosclerosis (FSGS) (21%) and nephrotic syndrome (36%). Thrombophilia screens were routinely undertaken in all children undergoing AVF formation. No evidence of thrombophilia was detected in the study population and consequently anticoagulation therapy was not used in the study’s patients. All patients with a functioning AVF were given aspirin prophylactically.

### Clinical indications for intervention and lesion anatomy

Low volume flow (27%) and multiple stenoses with poor quality dialysis^1^ (20%) were the principal indications for endovascular intervention. Mixed indications were seen in 23% of patients, where there was more than one clinical problem reported (e.g. difficult cannulations and high venous pressures) (Table 2). Thirty procedures were performed in 19 AVFs, with 46 stenotic lesions being treated overall. The majority of lesions were detected in the anastomotic (35%) and mid-vessel (28%) segments of the AVFs (Table 3). Multiple lesions were seen in 27% of the fistulae treated. Repeated interventions were required in 39% of children. The mean number of procedures per child was 1.7 (SD 0.97). As some children had more than on AVF, the mean number of interventions per fistula was 1.4 interventions (SD 0.87). Repeat angiography and angioplasty were performed in all cases where AVF malfunction recurred within 3 months of the initial angioplasty, in order to maintain hemodialysis using an AVF and to avoid CVC insertion.

**Table 2:** Clinical indications for intervention

	*N*	%
Thrombosis	4	13
Low volume flow	8	27
Multiple stenoses with dialysis inadequacy	6	20
Difficulties needling	3	10
High venous pressure and prolonged bleeding	2	7
Mixed	7	23
Total	30

**Table 3:** Location of lesions treated

	*N*	%
Anastomotic	16	35%
Post-anastomotic	8	17%
Mid-vessel	13	28%
Cephalic arch	8	17%
Central	1	2%
Total	46	

### Endovascular technique

Endovascular access was obtained through the venous segment of the AVF in 97% of cases treated. The target lesions were approached in a retrograde fashion (towards the anastomosis) in 53% of cases. All interventions were performed under general anaesthesia in a hybrid endovascular suite. Ninety percent of interventions involved balloon angioplasties alone. One case required pharmaco-mechanical thrombectomy using an AngioJet™ (Boston Scientific) device and tissue plasminogen activator.

Low profile 0.018 in. wires with 4Fr sheaths were commonly used. Angioplasty balloons were chosen according to the target vessel size. Prior to the intervention, 80 IU heparin/kg was given. Vasospasm was prevented by slowly inflating low pressure angioplasty balloons, maintenance of normotension during general anaesthesia and creation of a hypervolemic state using intravenous fluid boluses. If vasospasm did occur, the preferred approach was to wait for spontaneous resolution. Failure of vasospasm to resolve with conservative methods was addressed by intravascular administration of nitrates.

### Outcomes

Radiological success was defined as ability to undergo successful haemodialysis through a patent AVF immediately following endovascular intervention. This was achieved in 86% of cases (*n* = 25). The interventional failure rate was 14% (*n* = 4). This occurred in four patients, three of whom had multiple stenotic lesions. In all of these cases, there was at least one lesion which was either occluded or featured greater than 90% stenosis. Attempts at thrombectomy failed in one case, whilst the target lesion could not be crossed in two other cases. In the two cases where the lesions could not be crossed, surgical revision was undertaken: the first case involved proximalisation of a vein with post-anastomotic stenosis; the second case involved transposition of the cephalic vein for cephalic arch syndrome.

#### Complications

One patient experienced a rupture of the venous segment of the AVF following angioplasty of multiple lesions. This could not be controlled by balloon tamponade alone, requiring deployment of a covered stent. This controlled the extravasation but the AVF failed. This case represents the only significant immediate interventional complication (3%).

#### AVF stenosis outcome

The mean pre-angioplasty luminal stenosis (measured by comparison with adjacent normal vessel diameter) was 69% (SD 13.6). Post-angioplasty the mean luminal stenosis was 30% (SD 13.0). In the cases where flow measurement^2^ was available (*n* = 7), a mean improvement of 227 ml/min (SD 227) was noted followed endovascular intervention. This represents a mean 33% improvement in flow. Repeat angioplasty was necessary in 34% (*n* = 6) of patients. The mean time to re-angioplasty was 278 days (SD 234).

#### Dialysis quality 3 months post intervention

The mean URR was 76.6% (SD 3.1) and Kt/V was 1.63 (SD 0.33). In addition, the mean serum level of haemoglobin was 114.5 g/l, corrected calcium was 2.49 mmol/l, phosphate was 1.44 mmol/l and albumin was 38.9 g/l at 3 months following endovascular intervention.

## Discussion

Almost all children on a dialysis programme will be eligible for transplantation. Hence dialysis in childhood is seen as a ‘bridge’ until transplantation is possible. Currently, in the UK about 30% of children will experience haemodialysis before transplantation and the waiting time for first cadaveric renal transplant is approximately 1 year [10]. ‘Maintenance’ of dysfunctional AVFs is part of the natural history of managing vascular access and works in parallel with a surveillance programme. In adults, endovascular intervention is generally recognised as the preferred initial maintenance tool, but little is written about its role in children who have smaller AVFs and highly reactive vessels.

The indication for intervention was normally identified through a surveillance programme, unless the AVF had become acutely dysfunctional. Our current practice is for all children to undergo a 4-monthly surveillance assessment which facilitates early detection of AVF pathology. This involves an ‘ABCDE’ assessment including dialysis adequacy, volume flow measurement and clinical review (Table 4). In addition, we encourage an ‘open door’ policy for any concerns from the child/family or dialysis staff. This allows referral into a dedicated vascular access clinic with surgical, dialysis and ultrasound expertise. Families and referring healthcare staff are encouraged to screen for AVF-related complications using a clinical review checklist tool described in Table 5.

**Table 4:** Structured ‘ABCDE’ surveillance assessment of AVFs

Item	Clinical feature
Adequacy of dialysis	Urea reduction rate, Kt/V, blood pressure control, biochemistry
Blood flow rate	Measured in ml/min by using duplex ultrasonography or transonic flow monitoring device during dialysis
Clinical problems	Difficulties with dialysis reported by the patient, family or nursing staff, e.g. prolonged bleeding after decannulation
Diagnostic imaging	Duplex ultrasonography for peripheral vessels. MR or percutaneous angiography for central vessels
Examination	Physical examination of the AVF and limb

*AVF* arteriovenous fistulae

**Table 5:** Checklist for clinical complications of AVFs

Item	Clinical feature
Aneurysms	Abnormal dilatation of the arterial or venous components of the AVF
Black spots	Skin necrosis over the AVF with associated risk of bleeding and infection
Cellulitis	Erythema or discharge from the AVF
Distal ischaemia of the hand	Evidence of steal syndrome or distal embolisation in the hand
Extravasation	Evidence subcutaneous blood extravasation or ‘blow-out’ from the AVF manifested as bruising, haematomas or pain
Flow	Abnormal flow, detected by changes in the thrill and bruit
Girth	Upper limb soft tissue swelling/oedema demonstrated by change in arm diameter; indicative of central venous occlusion/stenosis
Hypertrophy	Upper limb hypertrophy caused by chronic hyperdynamic state

*AVF* arteriovenous fistulae

Our experience suggests that endovascular techniques are a valuable ‘first line’ tool for the maintenance of paediatric AVFs. Most cases can be performed as day case procedures with the child being prepared as an outpatient during haemodialysis and admitted from home on the day of the procedure for intervention under general anaesthesia. Immediately following intervention, the child should have a ‘test dialysis session’ and if successful be discharged under supervision. Rapid turnaround is important, as many children travel significant distances and minimising their school disruption is important. A high success rate can be anticipated (86% in this study) with excellent dialysis metrics at 3 months post procedure.

Most children will rotate through a ‘Dialysis – Transplant – Dialysis’ cycle before reaching adulthood. Hence, AVF maintenance is essential even in those with an imminent transplant as the functioning AVF can be subsequently used for phlebotomy following transplantation, by suitably experienced staff. Additionally, the AVF remains viable for future dialysis and the venous reserves are preserved for the future. It should also be noted that loss of AVF in the post-transplant period due to thrombosis is frequent [11]. Possible causes of AVF failure include hypovolaemia, pro-thrombotic immunosuppression and inexperienced phlebotomy. This should be guarded against with maintenance of a euvolemic state and training of phlebotomy staff in AVF management. Additionally, we recommend the prescription of 1 mg/kg oral aspirin for all children with an AVF.

Clinicians should be aware of any anticipated date for transplantation, which is helpful in planning a maintenance strategy for a malfunctioning AVF. In the UK, most living donation is performed within 3–6 months from initiating donor work-up, whilst the first deceased-donor transplant waiting time is approximately 1 year [10]. Hence, ‘nursing’ a functioning AVF with repeated endovascular interventions is preferable to abandoning it and electing for a CVC, especially if a transplant is on the horizon. Even modest improvements in luminal diameter and volume flows are worth achieving and often allow successful dialysis from a previously malfunctioning AVF. In our experience, endovascular intervention has a low risk profile (3%) and any resulting complications (e.g. extravasation, rupture or embolism) can usually be managed by additional endovascular techniques.

Failure to rescue a malfunctioning AVF using endovascular therapy (~ 14%) is most commonly seen in AVFs with multiple lesions. In such cases, surgical intervention may be a viable option in order to avoid the use of a CVC. In reality, this requires urgent coordination between the vascular access surgeon, anaesthetist and emergency theatres. The pathway of least resistance is often to defer to a CVC, but this should be resisted. The dialysis access nurse coordinator plays a pivotal role in prioritising such cases. When undertaking surgical revision, care should be taken in pre-operative planning of how to deliver dialysis post-operatively. Establishing new needling sites away from any areas of post-operative swelling and using single needle techniques may allow continued use of the AVF without introducing a CVC. If the child is self-needling on a home haemodialysis programme, it may be prudent to transfer back to in-centre dialysis with experienced dialysis staff until two permanent needling sites can be re-established. Compared with a surgical revision, minimally invasive interventions are associated with less pain, better cosmesis, shorter duration of hospitalisation and greater chance of avoiding a CVC.

One of the major advantages of an ‘endovascular-first’ approach to AVF malfunction is the avoidance of a CVC. CVCs have been shown to offer inferior dialysis adequacy, greater infection risk and higher hospitalisation rates compared to AVF-based haemodialysis [6] in children. Avoiding a CVC is also vital in preserving the central venous system for future AVF formation in children on chronic haemodialysis. However, currently available databases suggest this recommended practice is not routinely observed [4, 5]. In our experience, once a CVC is inserted there is often resistance from the child or parent to re-establishing dialysis using an AVF. Even the use of temporary dialysis catheters for short durations is not risk-free. These cause damage to central veins and increase risk for insertion-site and systemic infections. Temporary CVCs placed in the neck veins may negatively impact on future AVF formation in the upper limbs. When placed in the lower limbs, they may damage the femoral and iliac veins, making future renal transplantation more complex. Hence, AVF malfunction should be prioritised and follow an ‘emergency protocol’ for urgent assessment and rapid access to endovascular intervention where necessary.

The authors recognise that the study’s data is limited by the small population size and its retrospective nature, which did not allow sub-group analysis and measurement of long-term outcomes data.

## Conclusion

The management of paediatric AVF dysfunction with an ‘endovascular-first’ approach provides an effective solution for maintaining and rescuing vascular access in children on haemodialysis, with few associated complications. Repeated endovascular interventions may be necessary to avoid CVCs, until renal transplantation is possible. We recommend the development of emergency protocols and checklists for managing malfunctioning AVFs.

## Footnotes

1 Poor quality dialysis was defined by sub-optimal Urea reduction and Kt/v ratios

2 Measured by duplex ultrasound off dialysis
